# Domina Blackwell at Birmingham

**Published:** 1849-12

**Authors:** 


*Domina Blackwell at Birmingham.*—This lady, says the *London Times,* attended the Queen's Hospital, at Birmingham, and witnessed the operation of amputation of the thigh.

